# Tropomyosin-1 acts as a potential tumor suppressor in human oral squamous cell carcinoma

**DOI:** 10.1371/journal.pone.0168900

**Published:** 2017-02-09

**Authors:** Hao Pan, Liqun Gu, Binjie Liu, Yiping Li, Yuehong Wang, Xinna Bai, Long Li, Baisheng Wang, Qian Peng, Zhigang Yao, Zhangui Tang

**Affiliations:** 1 Department of Oral & Maxillofacial Surgery, Xiangya Stomatological Hospital & School of Stomatology, Central South University, Changsha, Hunan, China; 2 Department of Periodontics, Xiangya Stomatological Hospital & School of Stomatology, Central South University, Changsha, Hunan, China; 3 Department of Prosthodontics, Xiangya Stomatological Hospital & School of Stomatology, Central South University, Changsha, Hunan, China; 4 Department of Conservative Dentistry & Endodontics, Xiangya Stomatological Hospital & School of Stomatology, Central South University, Changsha, Hunan, China; 5 Department of Oral Pathology, Xiangya Stomatological Hospital & School of Stomatology, Central South University, Changsha, Hunan, China; China Medical University, TAIWAN

## Abstract

It is widely accepted that oral squamous cell carcinoma (OSCC) is a major contributor to the incidence and mortality of neck and head cancer. Tropomyosin-1 (TPM1), which is expressed at a low level, has been considered a prominent tumor-suppressing gene in a variety of solid tumors, although the precise mechanism of the TPM1 gene in OSCC progression remains unknown. We found that TPM1 expression levels decreased in OSCC patients and OSCC cell lines. The overall and cancer-specific survival of patients who exhibited low TPM1 levels were inferior to those of patients who had high TPM1 levels. It was also found that OSCC patients who suffered from disease stageⅠ-Ⅱ were more likely to have an up-regulated TPM1 expression level, and OSCC patients with lymph node metastasis had a higher probability of exhibiting reduced TPM1 expression. We show that overexpression of TPM1 can promote cell apoptosis and inhibit migration. Our results suggest that TPM1 can suppress tumors in OSCC, and the TPM1 expression level is related to OSCC patient prognosis.

## Introduction

Oral squamous cell carcinoma (OSCC) causes a portion of head and neck carcinomas, which may present as a primary lesion in any portion of the oropharynx or oral cavity. It was revealed by the International Agency for Research on Cancer that, in 2012, the number of new cases of OSCC and related deaths was 300,373 and 145,353, respectively[1]. Oral squamous cell carcinoma (OSCC) acts as a major cause of mortality and morbidity in patients suffering from neck and head cancer[2]. Despite extraordinary advances in many areas, such as detection, localized diagnosis and disease treatments, the overall survival rate of OSCC has only increased by 5% during last two decades[2–5], and the long-term survival rate of OSCC patients remains unfavorable. Consequently, new therapies, such as molecular therapies, for OSCC that can provide additional insights in disease screening and treatment selection are urgently needed, although the precise mechanisms of OSCC progression are still unclear.

The TPM family has approximately 40 different isoforms. Non-muscle cells express TPMs that have either a high or low molecular weight, including TPM1, TPM2, and TPM3 [6]. It was proposed that TPM1 is a crucial tumor-suppressing gene, which exhibited a low expression level in many solid tumors [7–15]. The tumor suppressing function of TPM1 was demonstrated in a breast cancer model [13]. Meanwhile, elevated expression levels of TPM1 can induce apoptosis and inhibit invasion in renal cancer cells [7]. The TPM structure protein family is among the most widely investigated tumor-associated protein families and plays an essential role in modifying the actin cytoskeleton and altering stress fibers. Actin cytoskeleton modification and stress fiber alteration are closely related to tumor-specific alterations of actin filament organization. Some evidence has indicated that the motility and invasiveness of tumor cells is increased by disrupted stress fibers, as well as by related adhesive structures mediated by high molecular weight TPMs [16–19]. Additionally, it was also shown that alteration in the actin cytoskeleton, mediated by high molecular weight TPMs, is related to the control of cell proliferation and motility. Previous studies have demonstrated that modification of microfilament structure was associated with cellular tumorigenicity and anchorage-independent growth [17–19]. Therefore, it is of prime importance to understand the effect of TPM1 on the actin cytoskeleton for development of new OSCC therapies.

TPM1 expression in tongue cancer has been reported in similar studies [20]. It was found that, as a potential target of microRNA 21, TPM1 plays a vital role in tongue carcinoma by enhancing apoptosis of cancer cells. However, tongue carcinoma is a type of OSCC, which includes tongue carcinoma, mouth floor carcinoma, buccal mucosa carcinoma, gingival carcinoma, lips carcinoma and oropharynx carcinoma [21]. Consequently, it is still unknown whether TPM1 functions in the same way in OSCC as it did in tongue carcinoma. Moreover, it was verified that TPM1 plays an essential role by inhibiting migration in colorectal carcinoma[22], breast cancer[23], gliomagenesis[12] and renal carcinoma[7], but none of these cancers were mentioned in Jinsong Li’s study[20]. Finally, research[20] has shown that TPM1 expression in tongue squamous cell carcinoma is down-regulated. However, in this study, the researcher transfected TPM1-siRNA in tongue squamous cell carcinoma cell lines, which decreased the expression of TPM1, and the results indicated that there was no influence on cell survival or cell growth. In our opinion, TPM1 is a tumor-suppressing gene, and enhanced TPM1 expression in tumor cell lines is needed. Most OSCC patients exhibited local advanced metastasis or lymph node metastasis at the time of diagnosis. Despite numerous anticancer treatments, the prognosis of OSCC patients is still unsatisfactory.

Consequently, there is an urgent need to clarify the role of TPM1 in OSCC, the mechanisms of TPM1 regulation and the precise molecular mechanisms involved in growth, invasion, metastasis and apoptosis of OSCC to optimize present clinical therapies.

The aim of this study was to determine whether TPM1 expression is associated with OSCC and prognosis, and if so, what role TPM1 plays in which aspect of tumor biology.

## Materials and methods

### Clinical specimens

The specimens, including seven fresh-frozen OSCC specimens and 7 OSCC adjacent normal epithelium specimens, from 7 OSCC patients who suffered from tongue carcinoma, buccal mucosa carcinoma and gingival carcinoma were obtained from the Oral and Maxillofacial Surgery Department of Xiangya Stomatological Hospital, Central South University from April 2013 to April 2014. Another batch of specimens, including 87 OSCC tissue specimens, which were fixed by formalin and embedded by paraffin, and 16 OSCC adjacent normal epithelium specimens, 87 OSCC patients diagnosed with tongue carcinoma, mouth floor carcinoma, mouth mucous membrane carcinoma, gingival carcinoma and oropharyngeal carcinoma were acquired from the Oral Pathology Department of Xiangya Hospital, Central South University from January 2010 to March 2011. No patients were given radiotherapy or chemical therapy before biopsy. Long-term survival analyses were conducted on all 87 patients and follow-up information, including overall survival and oral cancer-specific survival, was also collected. The clinical data, such as sex, histological grade, tumor class, disease stage, distant metastasis, recurrence and lymph node metastasis, of 87 patients were obtained from pathological and clinical records. The ethics committee of Xiangya Stomatological Hospital of Central South University authorized the protocol of the present study.

### Ethics statement

The study was conducted with the permission of the medical ethics committee of Xiangya Stomatological Hospital, Central South University. Written, informed consent was acquired from all subjects or guardians prior to using their resected specimens.

### Culturing cells

OSCC cell lines SCC15 and SCC25 were bought from American Type Culture Collection (ATCC, Manassas, VA, USA). HaCat cells were obtained from the Institute of Basic Medical Sciences, Chinese Academy of Medical Sciences (Beijing, China). Dulbecco's modified Eagle's medium/F12 (Gibco, Carlsbad, CA, USA) was used to culture SCC15 and SCC25 cell lines and Dulbecco’s modified Eagle’s medium (Gibco) was used to culture the HaCat cell line. Fetal bovine serum (10%, Gibco), 1,000 U/ml penicillin and 500 μg/ml streptomycin were added into all cell culture media, and cells were maintained in a humidifying incubator with 5% CO_2_ at 37°C.

### RT-PCR assay

TRIzol Reagent (Invitrogen, Carlsbad, CA, USA) was applied to isolate total RNA from treated cells, and then, a RervertAid FirstStrand cDNA Synthesis Kit (Thermo Scientific, Glen Brunie, MA, USA) was used to synthesize cDNA. An ABI 7500 Real-time PCR System, accompanied by SYBR Green detection (Life Technologies, Austin, TX, USA), were used to conduct real time PCR, based on the standard protocol given by the manufacturer’s instructions and with GAPDH as the internal reference. The primers of GAPDH were as follows: forward 5'-ATTCCATGGCACCGTCAAGGCTGA-3' and reverse 5'-TTCTCCATGGTGGTGAAGACGCCA-3'. The primers of TPM1 were as follows: forward 5'-GCCGACGTAGCTTCTCTGAAC-3' and reverse 5'-TTTGGGCTCGACTCTCAATGA-3'. The PCR reaction mixture was heated to (and maintained) at 95°C for five minutes, then maintained at 95°C for twenty seconds, cooled down to 60°C for thirty seconds, and heated to 72°C for thirty seconds, through forty cycles. Finally, it was cooled to 72°C for ten minutes. The expression level of the internal reference GAPDH was standardized to calculate the threshold cycle [24] of TPM1 and the relative quantities of TPM1 in the samples.

### Western blotting assay

The total protein of each sample was harvested using RIPA lysing solution (Thermo Scientific, Rockford, IL, USA), which contained Protease Inhibitor Cocktail (Thermo Scientific). A BCA protein assay kit (Thermo Scientific) was utilized to determine protein concentration, and proteins were isolated with SDS PAGE and then transferred onto a PVDF membrane (Millipore, Billerica, MA, USA). Subsequently, Tris buffer, which contained 0.1% Tween-20 and 5% skimmed milk, was used to block the membrane at 4°C overnight. Antibodies, including anti-TPM1 (RabMAb1:5000, Abcam, Catalog Number ab109505, Cambridge, MA, USA) and anti-GAPDH (1:5000, Bioworld, Catalog Number AP0060, Minneapolis, MN, USA), were used to incubate the membranes and then respective secondary antibodies (horseradish peroxidase-conjugated, 1:50000, Bioworld, Catalog Number BS13278, Minneapolis, MN, USA), conjugated with horseradish peroxidase, were used to bind the first antibodies. The results were obtained using an ECL detection system (Thermo Scientific), according to the instructions of the manufacturer.

### Immunohistochemistry assay

The antigen retrieval was conducted using a microwave method, and then, TPM1 antibody (1:500, Abcam) was added for immunostaining. After being incubated with biotinylated secondary antibody, the specimens were transferred to a DAB detection system. Hematoxylin was used to counterstain nuclei, according to the manufacturer’s protocol (DaKo, Glostrup, Denmark).

The visual evaluation of immunostaining degree was based on a four grade scoring system (ranging from 0–3). If the proportion of cells with positive staining was less than five percent, the score was zero. If the proportion was between five percent and twenty-five percent, the score was one. If the proportion was between twenty-five percent and fifty percent, the score was two. If the proportion was higher than fifty percent, the score was three. Each sample, which contained approximately 500 cells, was observed by two pathologists, and then, the average count was calculated based on the observation values. Clinical samples were divided into two groups, a high- expression group and a low-expression group. The high-expression group contained clinical samples with staining scores of 2 or 3 points. Conversely, the low-expression group contained clinical samples with staining scores of 0 or 1 points.

### Transfection assay

Trypsin was used to digest cells, which were then counted and seeded in 6-well plates, until the cell confluence reached 70%. Lipofectamine 2000 (Life Technologies, Carlsbad, CA, USA) was used to transfect OSCC cells (including SCC15 and SCC25) with M02-TPM1 plasmids and control (M02-con) plasmids (Catalog Number EX-E1174-M02, GeneCopoeia Company, Rockville MD, USA) to establish the TPM1-transfected and non-transfected cells. The efficiency of transfections was tested with western blotting and RT-PCR.

### MTS assay

SCC15 and SCC25 cells were transfected with M02-TPM1 and corresponding control plasmids for 24 h. Cell proliferation values were evaluated by a CellTiter 96^®^AQ_ueous_ One Solution Cell Proliferation Assay kit (Promega, Madison, WI, USA), following the guides recommended by the manufacturer. Cells were seeded into 96-well plates, after being digested and counted, at a density of 2x10^3^cells/well (0.2 mL/well). At 0 h, 24 h, 48 h, and 72 h after culture, 20 μL of MTS was added into every well, and the cells were incubated at a temperature of 37°C for two hours. Finally, the absorbance at 490 nm of each well, which represented cell quantity, was recorded with a microplate reader (BioTek Synergy2, Winooski, VT, USA) for the purpose of plotting growth curves.

### Transwell assay

SCC15 and SCC25 cells were transfected with M02-TPM1 and corresponding control plasmid for 24 h. A Cell Invasion Assay Kit (BD Biosciences, Billerica, MA, USA) was used to calculate and evaluate cell invasion, according to the instructions recommended by manufacturer. In short, 24 hours after transfection and after ECMatrix™ gel was precoated in the chambers, 4x10^4^ cells in 200 μL of serum-free culture medium were added into the upper chamber (Corning Costar, Tewksbury, MA, USA), while 0.5 mL of medium, which contained 10% fetal bovine serum, was put in the lower chamber as chemotactic factor. Subsequently, cells were incubated at a temperature of 37°C for 24 hours, with the crucial step of removing non-invading cells with cotton swabs. Then, cells that moved to the inferior membrane surface were permeated with one hundred percent pre-chilled methanol and dyed with 2% Giemsa staining reagent. Finally, the dyed cells were observed under random microscopic fields to minimize deviation. The cells in one hundred magnified fields were counted and the average value was calculated.

### Annexin V assay

SCC15 and SCC25 cells were transfected with M02-TPM1 and corresponding control vector for 24 h. An Annexin V-FLUOS staining kit (Roche, Nonnenwald, Penzberg, Germany) was used to collect and stain cells to evaluate cell apoptosis. A FACS flow cytometer (Becton-Dickinson) was used to analyze sample cells. Cells that exhibited Annexin V+ and PI- staining were regarded to be in the early stage of apoptosis, while cells that exhibited Annexin V+ and PI-/+ were regarded to be in the late stage of apoptosis. FlowJo software was used to analyze the results (Tree Star, Ashland, OR, USA).

### Statistical analysis

SPSS (version 21.0, IBM, Chicago, IL, USA) was used to process all statistical analyses at an alpha level of 0.05. Student’s t-test was applied for calculating the difference between 2 groups, while one-way ANOVA was used for more than three groups. Kaplan-Meier estimate and Log-Rank test were applied for evaluating the survival and Logistic Regression was utilized for assessing risk factors. All graphs were drawn by GraphPad Prism 6 software (GraphPad Software Inc., San Diego, CA, US).

## Results

### 1. Low expression of TPM1 in OSCC

**Fig 1:**
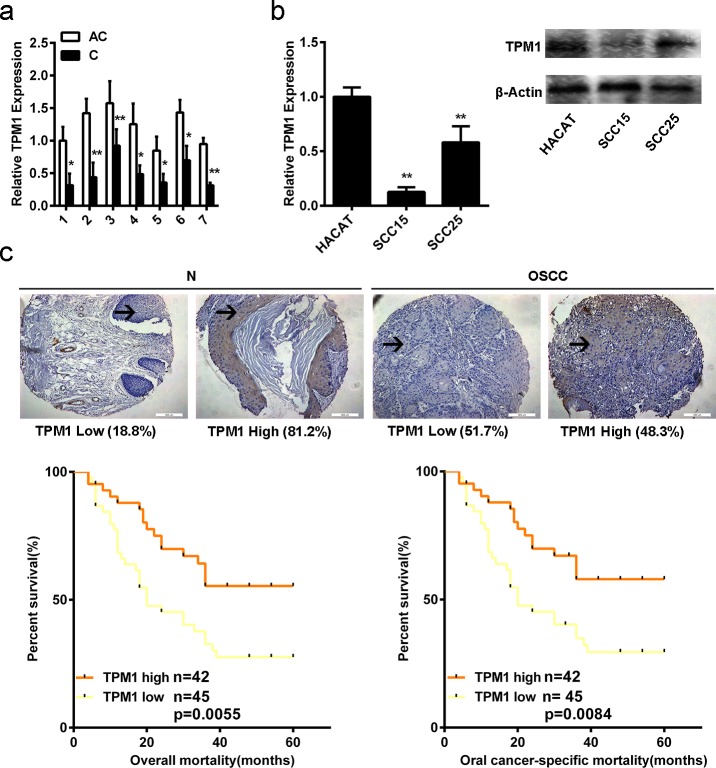
TPM1 expression pattern in OSCC. (a) The TPM1 mRNA level in OSCC and OSCC adjacent normal tissues from 7 patients was measured using a real-time PCR assay (Data are expressed as the mean±standard deviation. Data were obtained from triplicate assays; *p<0.05; **p<0.01). (b) Western blotting and RT-PCR were applied for evaluating the protein and mRNA levels of TPM1 in OSCC cell lines and a keratinocyte cell line (Data are expressed as the mean±standard deviation. Data were obtained from triplicate assays; *p<0.05; **p<0.01). **(c)** Representative images of TPM1 protein levels detected by immunohistochemical staining in OSCC tissue samples (N: OSCC adjacent normal tissue) and in OSCC tissues of patients who had follow-up information. Kaplan-Meier analysis estimated oral cancer-specific survival and overall survival, according to the TPM1 levels in OSCC patients.

An RT-PCR assay was utilized for determining the expression level of TPM1 in OSCC tissue and coupled adjacent normal tissue from each patient. The mRNA levels of TPM1 in OSCC tissue samples were notably inferior to that of adjacent normal tissue samples (p<0.05, Fig 1A).

RT-PCR and western blotting were utilized for determining the TPM1 levels in OSCC cell lines (SCC15, SCC25) and a normal epithelium cell line (HaCat). The mRNA, as well as protein levels, of TPM1 were distinctly and significantly different between OSCC and normal epithelium cell lines (p<0.05, Fig 1B, S1 Fig).

TPM1 expression levels in 87 patients (including 16 cases of OSCC adjacent tissues and 87 cases of OSCC tissues) were evaluated by immunohistochemistry. TPM1 was primarily expressed in epithelia cytoplasm rather than the nucleus or cytoskeleton. We observed that more OSCC tissues presented with down-regulated TPM1 than adjacent normal tissues (Table 1), and the difference was significant (51.7% vs. 18.8%; p<0.05, Fig 1C).

**Table 1:** TPM1 staining scores in OSCC.

Staining scores	0	1	2	3
OSCC	22	23	40	2
OSCC adjacent	1	2	12	1

### 2. Lower TPM1 expression was significantly related with OSCC progression

Overall survival and OSCC cancer-specific survival of the OSCC patients were associated with TPM1 expression. Eighty-seven patients were followed up and two patients passed away for unrelated reasons. One patient passed away due to apoplexy 33 months after surgery. Another patient passed away after a myocardial infarction 34 months after the operation. Consequently, these two cases were excluded from the survival rate analysis specific to oral cancer but accepted as an observation of death for overall survival rate analysis. Seventeen patients (17/42, 40.5%) in the TPM1 up-regulated group and 34 patients (34/45, 75.6%) in the TPM1 down-regulated group passed away. The survival curves show that a high-level of TPM1 significantly improved five-year overall survival (59.5% vs. 31.1%; hazard ratio, 0.45; 95% confidence interval [CI], 0.25 to 0.77; p<0.01) and five-year cancer-specific survival (61.9% vs. 26.7%; hazard ratio, 0.44; 95% CI, 0.24 to 0.77; p<0.01) in OSCC patients, compared with those with low-expression of TPM1 (Fig 1C). It was demonstrated by these results that patients who exhibited low TPM1 levels had worse cancer-specific and overall survival rates than those who exhibited high TPM1 levels in the cohort.

The association between TPM1 expression level and clinical features of OSCC patients were also analyzed in this study. TPM1 expression status was systematically evaluated through immunohistochemical staining and associated clinical information. The correlations between TPM1 expression level and sex, histological grade, recurrence, disease stage, tumor class, lymph node metastasis and distant metastasis were analyzed. There were no significant differences in sex (p>0.05), histological grade (p>0.05), recurrence (p>0.05), tumor class (p>0.05) and distant metastasis (no statistical data) between patients who exhibited high or low TPM1 levels. However, there was a notable relevance between disease stage and TPM1 protein level (p<0.05, Table 2). The probability of exhibiting an up-regulated TPM1 level was higher in OSCC patients of disease stageⅠ-Ⅱ. Moreover, it was also revealed that lymph node metastasis was remarkably correlative with TPM1 expression level (p<0.01, Table 2). The probability of exhibiting an up-regulated TPM1 level was low in patients suffering from lymphatic metastasis.

**Table 2:** Analysis of the correlation between expression of TPM1 in OSCC and clinicopathological parameters.

Characteristics	TPM1(%)		χ^2^	p value	Odd ratio(OR)	95% CI
	high expression	low expression				
Expression			5.197	**0.039**	0.247	0.066–0.929
OSCC	42 (48.3)	45 (51.7)				
OSCC adjacent	13 (81.2)	3 (18.8)				
Sex			1.651	0.209	2.114	0.657–6.804
Men	37 (51.4)	35 (48.6)				
Women	5 (33.3)	10 (66.7)				
Histological grade			2.002	0.184	3.072	0.585–16.182
Well	40 (50.6)	39 (49.4)				
Poor/moderate	2 (25.0)	6 (75.0)				
Recurrence			1.001	0.322	1.773	0.571–5.500
Present	9 (60.0)	6 (40.0)				
Absent	33 (45.8)	39 (54.2)				
Disease stage			4.990	**0.029**	2.726	1.106–6.718
Ⅰ-Ⅱ	32 (54.2)	27 (45.8)				
Ⅲ-Ⅳ	10 (30.3)	23 (69.7)				
Tumor class			0.501	0.483	1.500	0.484–4.651
T1-T2	36 (50.0)	36 (50.0)				
T3-T4	6 (40.0)	9 (60.0)				
Lymph node metastasis			8.098	**0.007**	0.250	0.092–0.681
Present	7 (25.9)	20 (74.1)				
Absent	35 (58.3)	25 (41.7)				
Distant metastasis			/	/	/	/
Present	0 (0.0)	0 (0.0)				
Absent	42 (100.0)	45 (100.0)				

### 3. TPM1 suppressed the proliferation and migration of OSCC cells

**Fig 2:**
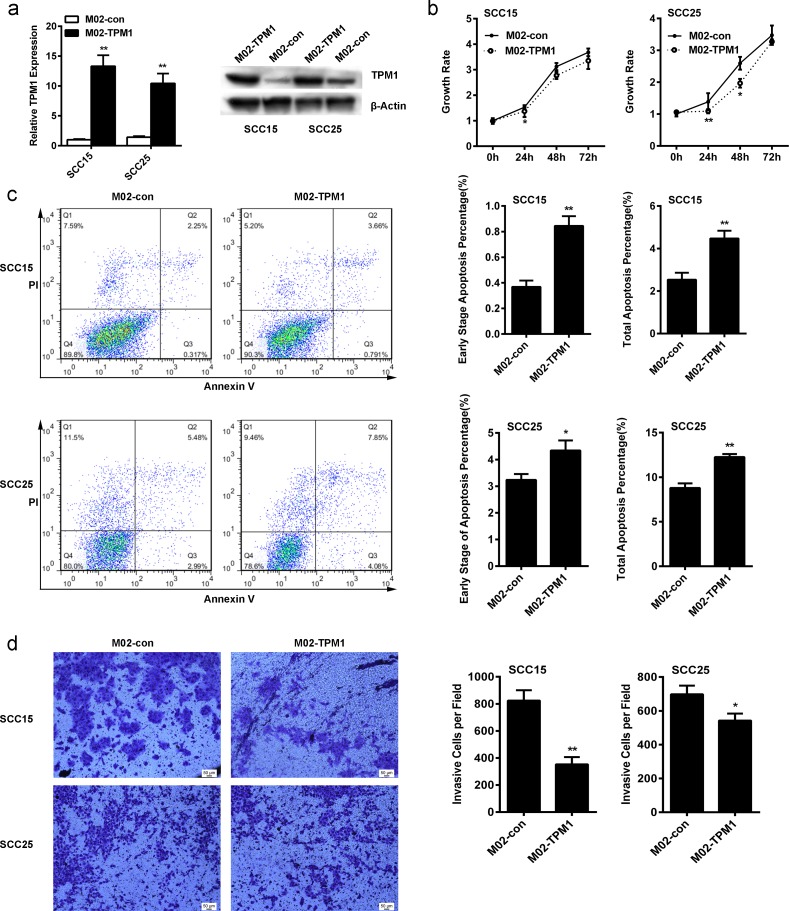
Tumor-suppressing functions of TPM1 in OSCC cells. The plasmid (M02-TPM1), which encoded TPM1, and empty plasmid were transfected into cells. **(a)** Western blotting and RT-PCR were applied to evaluate the protein and mRNA levels of TPM1 8 hours after the cells were transfected, separately. **(b)** The inhibitory effect of TPM1 on cell proliferation in SCC15 cells and SCC25 cells was evaluated by an MTS assay; the data were collected at 0 h, 24 h, 48 h, and 72 h (Data are expressed as the mean±standard deviation. Data were obtained from 6 distinct assays; *p<0.05; **p<0.01). **(c)** The promoting effect of TPM1 on cell apoptosis in SCC15 cells and SCC25 cells was evaluated by an Annexin V assay, and both early stage apoptosis and total stage apoptosis were promoted (Data are expressed as the mean±standard deviation. Data were obtained from triplicate assays; *p <0.05; **p<0.01). **(d)** The inhibitory effect of TPM1 on cell invasion in SCC15 cells and SCC25 cells was evaluated by a Transwell assay (Data are expressed as the mean±standard deviation. Data were obtained from triplicate assays; *p<0.05; **p<0.01).

Transiently transfected SCC15 and SCC25 cell lines, which expressed TPM1, were established to investigate the effect of TPM1 on human OSCC development and progress. RT-PCR and western blotting were conducted on SCC15 and SCC25 cells to analyze the expression levels of TPM1. It was revealed that TPM1 mRNA levels in cells transfected with M02-TPM1 plasmid were more than 10 times higher than that of cells with empty vectors (p<0.01, Fig 2A).

MTS cell proliferating assays were performed to assess the role of TPM1 in the growth of SCC15 and SCC25 cells after transfection (Fig 2B). After transfection, the SCC25 cell proliferation of the TPM1 group was reduced at 24 h (p<0.05), 48 h (p>0.05) and 72 h (p>0.05), in contrast to the negative control. In SCC25 cells, the proliferation of the TPM1 group was reduced at 24 h (p<0.05), 48 h (p<0.05) and 72 h (p>0.05), in contrast to the negative control after transfection. The results showed that, in OSCC cell proliferation, TPM1 does not have a significant effect.

SCC15 and SCC25 cells were transfected with M02-TPM1 or corresponding control plasmid for 24 h to evaluate the effect of TPM1 on induction of apoptosis. Flow cytometry was used to detect the cells that were stained with PI and Annexin V. The results confirmed that a high level of TPM1 could promote apoptosis in SCC15 and SCC25 cells (Fig 2C).

Twenty-four hours after SCC15 and SCC25 cells were transfected with TPM1, the rates of early-stage apoptosis (Annexin V+/PI-) were 0.845%±0.075% and 4.339%±0.379%, respectively, which significantly exceeded those of the negative controls (0.368%±0.049% and 3.238%±0.221% in SCC15 and SCC25 cells, respectively) (p<0.01 and p<0.05, Fig 2C). Moreover, the summarized rates of overall apoptosis stages (Annexin V+/PI±) after transfection with TPM1 were 4.475%±0.365% and 12.126%±0.170% in SCC15 and SCC25 cells, respectively, which significantly exceeded those of the negative controls (2.535±0.327 and 8.782±0.529% in SCC15 and SCC25 cells, respectively) (p<0.01 and p<0.01, Fig 2C).

SCC15 and SCC25 cells were transfected with M02-TPM1 or corresponding control plasmid for 24 h to assess the role of TPM1 in cell invasiveness.

The invasiveness of SCC15 and SCC25 cells transfected with TPM1 was obviously reduced (p<0.01 and p<0.05, respectively, Fig 2D). The number of invasive cells in SCC15-con and SCC15-TPM1 groups was 824±76 and 352±55, respectively. The number of invasive cells in SCC25-con and SCC25-TPM1 groups was 698±51 and 543±42, respectively. This was verified by a Transwell migration assay that showed that up-regulation of TPM1 could significantly inhibit the invasiveness of SCC15 and SCC25 cell lines.

## Discussion

Our study demonstrated that TPM1 could act as a tumor-suppressing gene in OSCC. By using real-time PCR, western blotting and immunohistochemistry, we detected TPM1 expression at both the mRNA and protein levels in OSCC cells and specimens from patients. We found that TPM1 expression levels were significantly higher in adjacent normal tissues than in OSCC lesions. In addition, TPM1 was much lower in OSCC cell lines than in a normal epithelium cell line. By comparing OSCC cell lines with different TPM1 expression levels, using an MTS assay, a Transwell assay, and an Annexin V assay, we found that high expression of TPM1 could slightly inhibit cell proliferation, strongly depress mobility and markedly promote cell apoptosis.

In our research, TPM1 expression was negatively related with certain clinical parameters, such as disease stage and lymph node metastasis, but positively related to prognosis. Consequently, it was presumed that a low TPM1 expression level in OSCC could be an independent risk factor of undesirable prognoses. In future studies, TPM1 levels in OSCC patients before and after treatment should be analyzed.

It was concluded, based on the above results, that TPM1 is a tumor suppressor in OSCC. The occurrence rates of lymphatic metastasis in patients who exhibited a low TPM1 level were higher than in patients with a high TPM1 level. Moreover, the prognosis was also positively correlated with TPM1 level.

Our results verified that TPM1 played a vital role in tongue cancer by inducing apoptosis in cancer cells [20]. It was confirmed that down-regulation of TPM1 expression was an early event in renal cancer cells. Low expression of TPM1 is associated with shorter disease-specific survival [7]. Additionally, it has been established that TPM1 is alternatively spliced to enhance cytoskeleton organization and terminal differentiation and inhibit malignancy in gliomagenesis [12]. Moreover, TPM1 has been shown to be notably reduced in pancreatic cancer patients and drug-resistant pancreatic cancer cell lines, and a low TPM1 level was correlated with worse survival [14]. It was reported that TPM1 expression was down-regulated in multiple cancers types, including renal, breast, esophageal, colorectal and ovarian cancer [6, 7, 23, 25–28]. Our findings on TPM1 in OSCC agreed with previous reports. In addition, we verified that TPM1 in OSCC not only induced apoptosis but also inhibited migration.

It is interesting that Zhi Wang’s study[29] showed a 6-fold increase in Tropomyosin 1 in OSCC when compared to precancerous oral leukoplakia. The reason for this phenomenon may be that tumor occurrence and development is a dynamic process, in which the expression of related genes changes dynamically. The expression of tumor-suppressor genes could rapidly decrease in a transient manner during the precancerous stage and finally recover in the advanced stage, which could present as an increasing trend in expression in comparison to the precancerous stage. Our data showed that TPM1 expression was lower in OSCC than in normal tissues and cells. It also indicated that the function should be studied from more angles and with more specimens. There has been a report that currently there is no evidence of a treatment that is effective for preventing oral leukoplakia from developing into oral cancer.[30] In this case, TPM1 may be a potential target to solve this problem. However, the regulation and mechanism of TPM1 in oral leukoplakia is still unknown. Therefore, more related research should to be performed in the future.

Another study focusing on OSCC and the tropomyosin family reported that expression of Tropomyosins 2 and 4 was increased in OSCC[31]. This result is consistent with previous reports in other solid tumors, such as the finding that TPM2 is highly expressed in breast cancer[32], colon cancer[33] and ovarian cancer[28], while TMP4 is highly expressed in lung cancer[34] and ovarian cancer[28, 35]. It has been revealed that there are abundant isoforms in the Tropomyosin family. Despite similar structures, isoforms exert distinct functions and behave differently in organisms.

In this study, it was revealed initially that TPM1 acted as a tumor suppressing gene in OSCC. Our data indicated that down-regulated TPM1 was a vital marker for poor prognosis in OSCC.

However, there were still limitations in this study. Primarily, it has been shown by various studies that microRNA-21 suppressed TPM1, and TPM1 is a target gene of microRNA-21[36–38]. As an oncogene, microRNA-21 was investigated in solid tumors and observed to be up-regulated in tongue carcinoma [20, 39], although no luciferase reporter assay results, which could be used to verify the association between miR-21 and TPM1, were presented in any of these studies. Consequently, it was inferred that microRNA-21 could regulate TPM1 expression while promoting OSCC. Future studies on genetic modifications, specifically in OSCC, are needed to further verify our observations. Second, since the sample size in this study (87 OSCC patients) was comparatively small, the results should be confirmed in larger-scale studies. Third, these conclusions were based on the responses of three cell lines and might not reflect the processes that occur in intact organisms. The next step we will take is to confirm our results in nude mice.

Our findings could be valuable for seeking potential markers of OSCC metastatic progression and promising molecular targets for treating OSCC.

In summary, it was demonstrated in this study that TPM1 acted as a tumor-suppressing gene in OSCC, and the TPM1 expression level was related to OSCC prognosis.

## Supporting information

S1 Fig (RAR)
